# COVID-19 and social distancing

**DOI:** 10.1007/s10389-020-01321-z

**Published:** 2020-05-25

**Authors:** Meirui Qian, Jianli Jiang

**Affiliations:** National Translational Science Center for Molecular Medicine, Fourth Military Medical University, Xi’an, 710032 People’s Republic of China

**Keywords:** COVID-19, Coronaviruses, Pneumonia, Social distancing

## Abstract

**Aim:**

COVID-19 has spread rapidly worldwide since it began, greatly affecting peoples' lives, social economies and medical systems. At present, little is known about the disease, and vaccines are still under development. Therefore, in the face of severe outbreaks, previous effective experience can help people better protect themselves and their families. The aim of this article is to discuss the social distancing measures for COVID-19.

**Subjects and methods:**

Literature and document search.

**Results:**

Recent research and a novel coronavirus pneumonia prediction model revealed social distancing measures and wearing masks are required to mitigate hospital system overload and prevent pathogen exposure. After a series of social distancing measures, there are 309 cities with zero cases and 34 cities with confirmed cases in China as of April 13, 2020.

**Conclusion:**

From China's experience with novel coronavirus pneumonia, we know that social distancing is the most effective measure at present. We need to win more time to allow limited medical resources to save lives.

## Introduction

China reported a novel coronavirus in Wuhan, Hubei Province, on December 31, 2019 (Gralinski and Menachery 2020). Wuhan is an urban town located in the central part of China. It is a significant transportation and business hub. Suspected and confirmed cases began to appear in various parts of the state. China shared the novel coronavirus pneumonia gene sequence in January 12, 2020. As of January 22, 2020, 571 cases of COVID-19 had been reported in 25 provinces (including districts and cities) in China (Lu 2020). The patients showed typical respiratory symptoms (such as fever, coughing, shortness of breath, and inflammatory lung infiltration) and other symptoms such as fatigue, myalgia, and diarrhea (Huang et al. 2020). Some cases were atypical or showed asymptomatic symptoms in this epidemic. On February 11, 2020, the World Health Organization (WHO) named this pneumonia Coronavirus Disease-2019 (COVID-19). As of April 13, 2020, novel coronavirus pneumonia cases were confirmed in 200 countries and regions worldwide. The case fatality rate (CFR) of COVID-19 was 2.3% (44/1023), much lower than that of SARS (10%) and MERS (36%) (de Wit et al. 2016; Wu and McGoogan 2020). Suspected COVID-19 patients (with symptoms) could be diagnosed by chest CT and polymerase chain reaction (PCR) kit. However, there were no specific effective antiviral drugs for treatment, and the vaccine was still in the experimental stage (Zumla et al. 2016).

## Strategies to combat the epidemic situation

Humans have a very old but extremely effective way to respond to sudden outbreaks of infectious diseases—isolation. The core of the infectious disease outbreak lies in its infectivity: it can be transmitted directly or indirectly from a person to one or more people (Li 2020). If a person who has the disease cannot spread it to more than one person, the disease will gradually disappear. Like other viruses such as SARS and MARS, although each infectious disease has its new characteristics, prevention and control involve three factors: the pathogen, transmission route and susceptible population. There are three core elements of isolation: find and manage the source of infection; cut off the transmission channels; protect vulnerable groups (Mikulska 2019).

In China, the state took decisive measures to implement medical isolation for patients and close contacts, block traffic, cancel public activities and require people to wear masks and frequently wash their hands. Strong control measures were implemented in cities such as Wuhan, where the source of infection was concentrated, while other provinces and cities also conducted careful screening and isolation of the exported cases. Interestingly, after this strategy was identified, the provinces and areas responded positively and quickly. The residential areas also formulated relevant travel systems, using social software to control the travel of each resident, and the flow of people between different areas was also strictly controlled. For example, one person from every family could go out every 2 days to purchase necessities; in public places, the distance between people in line needed to be > 2 m; express delivery and takeout also allowed contactless delivery. Research showed that social distancing measures were most effective when large-scale return to work took place in early April. This reduced the median number of infections by 92% (IQD 66–97) and 24% (IQD 13–90) in mid-2020 and by the end of 2020, respectively (Prem et al. 2020). This time, compared with the SARS outbreak in 2003, the situation was more complicated, including the time of the outbreak in the geographical location of Wuhan because of movement related to the Spring Festival, but highly effective control of the spread of the pandemic was shown in China.

## Social distancing

Social distancing involved keeping a distance of 1.5 m between people, which can prevent the spread of most respiratory infectious diseases. Social distancing is one of the most effective measures to reduce the spread of the virus, which is transmitted by air droplets. The droplets produced by coughing, sneezing or forced speaking have a certain transmission distance. By keeping this distance, we can reduce the spread of the virus. Wearing masks, washing hands frequently and disinfecting with alcohol also help to prevent the virus from spreading from one person to another. To control the disease, the World Health Organization recommended that countries should strengthen case detection, track and monitor contacts, practice isolation from close contacts and isolate cases as well as implement traffic control and suspend large gatherings. A novel coronavirus pneumonia prediction model was established by using the big data of the University of Washington Health Index and Evaluation Center (IHME) (IHME 2020). Analysis of the epidemic situation in seven locked down cities in Wuhan, Italy and Spain showed that maintaining social distancing really achieved results. Because these cities implemented lockdowns, the epidemic quickly reached a peak, so far not climbing again. Erin Mordecai, a biologist at Stanford University, and a team of researchers developed an interactive simulator to simulate the spread of COVID-19 over time, demonstrating the role of social isolation and social distancing in epidemic control (Erin 2020).

Notably, the government quickly adopted the correct strategy, e.g., social distancing, thus controlling the rate of the increase of cases and winning more time for doctors. If this had not been the case, it would have been impossible for medical staff from another provinces to go to Wuhan for support, and Wuhan appeared to be the only outbreak city. In terms of cost, social distancing also saves medical resources, such as masks, hand sanitizers, alcohol-based disinfectant, etc. This gives our healthcare professionals, hospitals and other institutions more valuable time to prepare, prevent the disease and help people who have been diagnosed with coronavirus. There were 50,633 confirmed cases in Hubei on February 18, 2020, because of a peak after the closure of the city. Then, the number of cases began to decline. As of April 13, the number of confirmed cases in Hubei Province was 244.

However, there are objections to social distancing. Thomas Abel reported that social distancing might lead to depression and anxiety in some people (Abel and McQueen 2020) and that social distancing measures are not appropriate in this situation because they will cause more panic and anxiety among people, which will also have an impact on social stability. Of course, this crisis has psychological impacts on patients, health care workers and the population. However, we should routinely provide psychological support instead of stopping social distancing measures (Kim and Su 2020).

## Conclusion

The way novel coronavirus pneumonia is transmitted means that taking certain social distance measures is the most effective practice to prevent and control the disease. WHO’s South East Asia Region director Poonam Khetrapal Singh found that we could reduce the virus transmission by following social distancing measures. The spread of this disaster is global. To fight the epidemic, people from all countries need to unite to see the coming of hope.
